# Sex‐dependent effects of a high‐fat diet‐induced obesity model on cerebrovascular health and brain metabolism

**DOI:** 10.1113/EP093187

**Published:** 2025-10-03

**Authors:** Nicole N. Eminhizer, Dena Lin, Alec Hanshew, Jackson Stewart, Steven Ball, Christa Lilly, Saina S. Prabhu, Kate Karelina, Eric E. Kelley, Randy W. Bryner, Dharendra Thapa, Paul D. Chantler

**Affiliations:** ^1^ Division of Exercise Physiology West Virginia University School of Medicine Morgantown West Virginia USA; ^2^ Department of Neuroscience West Virginia University School of Medicine Morgantown West Virginia USA; ^3^ Department of Pharmaceutical Sciences West Virginia University School of Medicine Morgantown West Virginia USA; ^4^ Department of Epidemiology and Biostatistics West Virginia University School of Medicine Morgantown West Virginia USA; ^5^ Department of Physiology and Pharmacology West Virginia University School of Medicine Morgantown West Virginia USA

**Keywords:** cerebrovascular health, lysine acetylation, obesity, sex dependent

## Abstract

Mid‐life obesity is a major risk factor for neurodegenerative diseases, with mitochondrial and cerebrovascular dysfunction considered key mediators. Lysine acetylation is a reversible post‐translational modification that regulates several mitochondrial metabolic and biochemical processes. The present study investigated the sex‐dependent effects of brain lysine acetylation and cerebrovascular and cognitive health in a high fat diet (HFD)‐induced obesity mouse model. We hypothesize that a HFD will cause an increase in acetylation, dysregulating mitochondrial respiration, potentially due to the decline in overall cerebrovascular health. Six‐month‐old C57/Bl6 mice (M/F) were placed on a 60% HFD or normal chow (CON) for 4 months. Changes in cerebral blood flux (CBF), behavioural testing, glucose tolerance testing and body composition were tested. Brain lysates were probed for various substrate utilizations, bioenergetics proteins and lysine acetylation. A HFD resulted in global metabolic dysregulation, with a substantial increase in weight and fat mass, with a greater increase in female mice; however, no cognitive changes were noted. Additionally, unlike female mice, males demonstrated a decrease in CBF after a HFD. Brain lysine acetylation was decreased in male HFD mice but increased in female HFD mice. Similarly, acetylation levels of fatty acid oxidation protein (long‐chain acyl‐CoA dehydrogenase), glucose oxidation proteins (pyruvate dehydrogenase, pyruvate carboxylase) and electron transport chain complex I (NDUFB8) and IV (MTCO1) proteins were decreased in male and increased in female brains after a HFD. In summary, our findings propose lysine acetylation as a novel and potential regulatory mechanism that impacts vascular and metabolic function in the brain mitochondria in a sex‐dependent manner.

## Abstract (highlights)

- **What is the central question of this study?**
  What are the sex‐dependent effects of brain lysine acetylation and cerebrovascular and cognitive health in a high fat diet‐induced obesity mouse model?
- **What is the main finding and its importance?**
  Lysine acetylation has a sex‐dependent role in regulating proteins of glucose and fatty acid oxidation and mitochondrial electron transport chain complexes. Lysine acetylation is a novel and potential regulatory mechanism that impacts vascular and metabolic function in the brain mitochondria in a sex‐dependent manner.

## 1 INTRODUCTION

The obesity epidemic continues to expand, leading to widespread metabolic dysregulation (Mitchell et al., 2011). Due to the high metabolic demand of the brain, this obesity‐induced metabolic dysregulation can have significant clinical implications for the brain (Camandola & Mattson, 2017; Kordestani‐Moghadam et al., 2020). We and others have shown that obesity leads to significant structural and functional changes to the cerebral arteries, resulting in a dysregulation of cerebrovascular health (Brooks et al., 2018, 2015). A reduction in cerebral blood flow (hypoperfusion) has been observed in obese patients (Alosco et al., 2012; Selim et al., 2008; Willeumier et al., 2011). However, sex‐dependent differences are noted in cerebral blood perfusion, with females having higher perfusion rates than males (Knight et al., 2021). Cerebrovascular impairment can lead to unmet nutrient demands, resulting in mitochondrial dysfunction and cognitive decline (Balasubramanian et al., 2021; Incalza et al., 2018). However, the mechanisms behind obesity‐induced brain hypoperfusion are not fully known (Toyama et al., 2015), with increases in reactive oxygen species (ROS) proposed as a potential mediator of brain, vascular and metabolic damage (Incalza et al., 2018; Tan & Norhaizan, 2019).

The mitochondria are a significant source of ROS and play a critical role in the brain by supplying the energy needed for brain health (Song et al., 2024). Glucose, the primary fuel substrate in the brain, undergoes glycolysis and is shuttled to the mitochondria as pyruvate to undergo oxidative phosphorylation (OXPHOS). Additionally, fatty acid oxidation (FAO) accounts for approximately 20% of the energy production in the brain (Ebert et al., 2003). Dysregulation of these energy sources has been found to cause mitochondrial dysfunction and lead to various neurological diseases, including Alzheimer's and Parkinson's disease (Abiose et al., 2024; Amelianchik et al., 2022; Anandhan et al., 2017; Christensen & Pike, 2017). The increase of free fatty acids (FFA), caused by a high‐fat diet (HFD), has been proposed as a possible agonist to insulin resistance seen within skeletal muscle (Ellis et al., 2000; Sears & Perry, 2015). However, the exact mechanism behind the insulin resistance is still unknown. Within the heart, increases in circulating FFA and alterations in FAO enzymes occur, leading to heart failure (Carley et al., 2007; Cole et al., 2011). In addition, post‐translational regulation of cardiac mitochondrial proteins by lysine acetylation regulates FAO in the heart of mice on a HFD (Thapa et al., 2017). The role of lysine acetylation in mitochondrial regulation is growing in significance as several mitochondrial enzymes are found to be acetylated, causing potential effects on function (Wang et al., 2010; Young et al., 2002). In the context of the brain, very little is known regarding the effect of FFA on metabolic regulation. Within the hypothalamus, control of the satiety hormone, leptin, and hormonal signalling of insulin are regulated (Thaler & Schwartz, 2010). Increases in palmitic acid‐enriched ceramides within the hypothalamus are evident with a HFD, potentially leading to insulin resistance (Borg et al., 2012). However, the effects of FFA on the whole brain have not been investigated. Specifically, there are no reported data, to our knowledge, exploring the role of lysine acetylation in the brain in regulating mitochondrial enzymes within a diet‐induced obesity model.

More recently, sex differences have been reported in the brains of mice whilst exposed to a HFD (Dreux et al., 2025; Lefebvre et al., 2024; Murtaj et al., 2022). The sex‐specific progression of weight gain and hyperglycaemia within a diet‐induced obesity mouse model depends upon the age of onset of the HFD (Salinero et al., 2018). Middle‐aged female mice show a more significant gain in weight and glycaemic levels than juvenile and young adult counterparts whilst on a HFD, whereas males have the highest metabolic deficits at the juvenile time point (Salinero et al., 2018). These data emphasize the importance of determining the sex‐specific actions of a HFD on brain health. Further, post‐translational phosphorylation and acetylation of various brain proteins are sex‐dependent (Qin et al., 2013). However, the sex‐dependent post‐translational modification with obesity and its role in brain, vascular and cognitive outcomes are unknown. This study aimed to explore the sex‐dependent effects of acetylation on the HFD brain. We hypothesized that a HFD would cause an increase in acetylation, dysregulating mitochondrial respiration, potentially due to the decline in overall cerebrovascular health. We further hypothesize that males will have more significant metabolic dysregulation than females on a HFD.

## 2 METHODS

### 2.1 Animal housing

All experiments were approved by the West Virginia University Health Sciences Center Animal Care and Use Committee through the guidelines given by the National Institutes of Health and the Office of Laboratory Animal Welfare. At 5 months of age (*n* = 32), male and female C57/BL6 mice (stock no. 000664) were obtained from The Jackson Laboratory (Bar Harbor, ME, USA). Mice were group‐housed at West Virginia University Health Science Center animal husbandry and had access to housing, food and water ad libitum. Mice were monitored regularly to ensure that they had sufficient water and food. In a few cases where all or most of the food was consumed before the next measurement time, food was replenished as needed, and amounts were recorded accordingly. The temperature in the husbandry was 20–24°C, and the humidity was 45–55%. The mice were exposed 12–12 h light–dark cycle. After acclimation, when mice were 6 months old, they were randomly assigned to specific groups by a blind researcher. Eight mice per sex per group were assigned to a HFD (60% fat, 20% protein, and 20% carbohydrate – 5.24 kcal/g; Research Diets, NJ, USA; D12492) or a standard chow diet (CON) provided by the animal facility (18% fat, 24% protein and 58% carbohydrate – 3.1 kcal/g, Teklad, WI, USA 2918). The CON and HFD lasted for 4 months till mice were 10 months of age to represent a middle‐aged time point as outlined by The Jackson Laboratory. Due to aggression, commonly seen in this strain, some HFD female mice were excluded from measurements due to injury.

### 2.2 Body weight and composition

Mice were weighed weekly, and lean, fat and water mass were measured at 9.5 months using an echoMRI (EchoMRI 100H Body Composition Analyzer, Tx, USA).

### 2.3 Glucose tolerance testing

After 5–6 h of fasting, mice were tested for fasting glucose using the ACCU‐CHEK Guide system. After the initial reading, mice received a glucose bolus (1.25 mg/kg) through an i.p. injection. Glucose levels were recorded at 20, 40, 60 and 120 min to measure the rate of glucose metabolism. The incremental area under the curve (iAUC) was reported after adjusting for baseline differences in fasting glucose.

### 2.4 Behavioural testing

Mice underwent open‐field testing for locomotion and anxiety‐like behaviour. They were placed in the Photobeam Activity System (CA, USA) Open Field apparatus for 30 min (6 phases lasting 300 s each). Locomotion and central tendency were calculated by beam breaks using San Diego Instruments (PAS) software. Y‐maze was then used for working memory. Mice were placed in the Y‐maze apparatus for a 5 min test. The testing period was then recorded and analysed using the Any Maze software.

### 2.5 Cerebral blood flux

All animals underwent cerebral blood flux (CBF) measurements using a RWD Laser Speckle Imaging System (RWD, RFLSI ZW, Sugar Land, TX, USA) at 10 months before terminal procedures. Mice were anaesthetized with 1.8% isoflurane and given lidocaine (0.5 mg/kg) and bupivacaine (1.5 mg/kg) subcutaneously under the skin of the skull, and meloxicam (5 mg/kg) was given between the shoulder blades. Hair was removed from the head, sterilized with ethanol and beta iodine, and then a 2.5 cm incision was made in the skin above the skull. Mineral oil was used to keep the skull moisturized (and to prevent skull drying that disrupts imaging), and four images of cerebral blood flow were taken at the following settings: processing mode; spatial algorithm; step mode; spatial filter, 15 s; interval, 1 min. These images were then analysed using RFLSI Analyzer software. Only images that were clear and matched the settings listed above were used for analysis, ensuring consistency and accuracy in measurements.

### 2.6 Tissue collection

Mice were euthanized under 4–5% isoflurane to induce anaesthesia, then maintained with 1.5% isoflurane whilst a thoracotomy was performed, and a blood sample was collected via cardiac puncture. Then the diaphragm was cut and the heart removed. At this point, the head was separated from the body and the brain removed from the skull (and processed as described below). The right hemisphere underwent a gross dissection and was flash frozen in liquid nitrogen to be processed later.

### 2.7 Circulating markers

Blood plasma was used to measure metabolic markers using Bio‐Plex Pro Mouse Diabetes panel to measure insulin and leptin (Bio‐Rad, Hercules CA, USA, model no. 171f7010M). Blood plasma samples were run in duplicate.

### 2.8 Protein isolation

Brain tissue was isolated and homogenized in a 1% CHAPS lysis buffer (1% CHAPS, 150 mM NaCl, 10 mM HEPES, pH 7.4). The lysate was placed on ice for 2 h and centrifuged at 10,000 *g* for 10 min. The supernatant was collected, and protein concentration was measured using Bradford plus protein assay following the manufacturer's instructions (cat. no. 23236, Thermo Fisher Scientific, Waltham, MA, USA). Brain lysates were then used for western blotting and co‐immunoprecipitation experiments.

### 2.9 Western blotting

For our western blot experiments, an equal amount of protein was utilized. Brain lysates were prepared using lithium dodecyle sulfate (LDS) sample buffer and reducing agent. A Bolt SDS/PAGE 4–12% or 12% Bis–Tris gel was used to separate proteins and transfer them to nitrocellulose membranes (all Thermo Fisher Scientific). Protein expression was measured using several different antibodies as listed below: rabbit acetyl‐lysine (cat. no. 9814s), rabbit glutamate dehydrogenase (GDH) (cat. no. D9F7P), rabbit pyruvate dehydrogenase (PDH) (cat. no. C54G1) from Cell Signaling Technology, Danvers, MA, USA; rabbit long‐chain acyl‐CoA dehydrogenase (LCAD; cat. no. 17526), rabbit hydroxyacyl‐CoA dehydrogenase trifunctional multienzyme complex subunit alpha (HADHA) (cat. no. 10758), rabbit pyruvate carboxylase (PC) (cat. no. 16588) from Proteintech, Rosemont, IL, USA; mouse total OXPHOS cocktail (cat. no. ab110413) to analyse NADH:ubiquinone oxidoreductase subunit B8 (NDUFB8), Succinate Dehydrogenase Complex, Subunit B (SDHB), ATP5A, mitochondrially encoded cytochrome c oxidase (MTCO1) and ubiquinol‐cytochrome c reductase core protein 2e (UQCRC2) from Abcam, Waltham, MA, USA. For our secondary antibodies, fluorescent anti‐mouse secondary red 680 nm (cat. no. 92668070), green 800 nm (cat. no. 92532210) or anti‐rabbit secondary antibodies red 680 nm (cat. no. 92668071), green 800 nm (cat. no. 92632211) from Li‐Cor (Lincoln, NE, USA) were used to detect expression levels. Finally, protein densitometry was measured using ImageJ software (National Institutes of Health, Bethesda, MD, USA).

### 2.10 Immunoprecipitation

For our acetyl‐lysine immunoprecipitation pulldown experiments, total brain lysates in 1% CHAPS buffer with deacetylase inhibitor (100 µM Trichostatin A and 5 mM Nicotinamidste) were used. An equal amount of protein was incubated overnight with rabbit acetyl‐lysine antibody, rotating at 4°C. Immunocaptured proteins were then isolated with Protein‐G agarose beads (Cell Signaling Technology) by incubating for 2 h at 4°C. After this incubation, it was washed multiple times with CHAPS buffer and eluted in LDS sample buffer by heating at 95°C. Samples were then separated using Bolt SDS/PAGE 4–12% or 12% Bis–Tris gels, as mentioned above, and appropriate primary antibodies were used. Protein densitometry was measured using ImageJ software.

### 2.11 Statistics

Given the difficulty of blinding in HFD studies (i.e., the colour of food and the size of mice), we limited bias by coding mice during the molecular experiments and data analysis (for CBF, behaviour and molecular tests) so that the research team was unaware of group allocations. Analyses were conducted using SAS 9.4 (SAS Institute, Cary, NC, USA) and GraphPad Prism (version 10.0.2, GraphPad Software, Boston, MA, USA). A three‐way ANOVA was run for body weight change over time by sex and diet with multiple comparisons. Glucose tolerance tests (GTT) were run across multiple time points. Total area under the curve (AUC) was calculated for each mouse on GTT using the trapezoidal rule. Outliers for all outcomes were checked using *z*‐scores, and any outliers found were excluded. Only one outlier was found and removed. This outlier was found within the Bio‐Plex Pro Mouse Diabetes panel, as one HFD female mouse had a more than 10× higher value than the average. This same experiment also included the exclusion of a CON female mouse that had undetectable values. Continuous outcomes (e.g., GTT incremental AUC, fat mass, lean mass, weight gain) were described using means and standard deviations. Next, two‐way ANOVAs (group by sex) with main and interaction effects were run using a general linear model (GLM) procedure, allowing for unequal sample sizes by group. Referent groups were set to ‘control’ and ‘male’. Planned comparisons in follow‐up to the group by sex interaction terms included within sex assessments.

## 3 RESULTS

### 3.1 Diet‐induced obesity measures and metabolic profile

Both sexes on a HFD had increased body weights compared to controls (Table 1). However, a sex‐by‐group interaction was identified (*P* = 0.028), whereby females after a HFD had a greater increase in body weight than males after a HFD (Figure 1). An HFD in male and female mice increased fat mass and decreased lean mass compared to controls (*P *< 0.05). However, a HFD in female mice resulted in greater fat mass and lean mass changes than in male mice (*P *< 0.05, Table 1). No differences in body weight, lean mass or fat mass were noted between male and female CON groups. Food consumption was higher in male and female HFD groups than in control groups, and no differences were noted between control males and females (Table 1).

**TABLE 1:** Characteristics of control (CON) and high‐fat diet (HFD) male and female (M/F) mice.

	CON M	HFD M	CON F	HFD F
Final weight gain (%)^†^	14.1 ± 7.9	55.3 ± 9.6^*^	13.9 ± 9.2	81.1 ± 29.5^*^
Fat mass (%)^†^	31.9 ± 6.4	39.4 ± 1.8^*^	28.7 ± 6.9	49.7 ± 5.6^*^
Lean mass (%)^†^	66.3 ± 5.4	59.2 ± 1.2^*^	69.5 ± 9.4	48.7 ± 5.3^*^
Weekly food consumption (g)^†^	23.0 ± 7.0	19.9 ± 6.1^*^	23.4 ± 7.7	17.9 ± 8.0^*^
Weekly food consumption (kcal)^†^	77.2 ± 21.7	103.2 ± 31.8^*^	72.4 ± 23.9	93.3 ± 41.6*

Values reported as means ± SD. CON M *n* = 8 per group, HFD M *n* = 8 per group, CON F *n* = 8 per group, and HFD F *n* = 5–8 per group (due to aggression between animals that led to injury). *P < 0.05 within sex (i.e., CON M vs. HFD M, CON F vs. HFD F); ^†^ *P* < 0.05 two‐way ANOVA interaction between diet and sex. CON, control; HFD, high‐fat diet; F, female; M, male.

**FIGURE 1:**
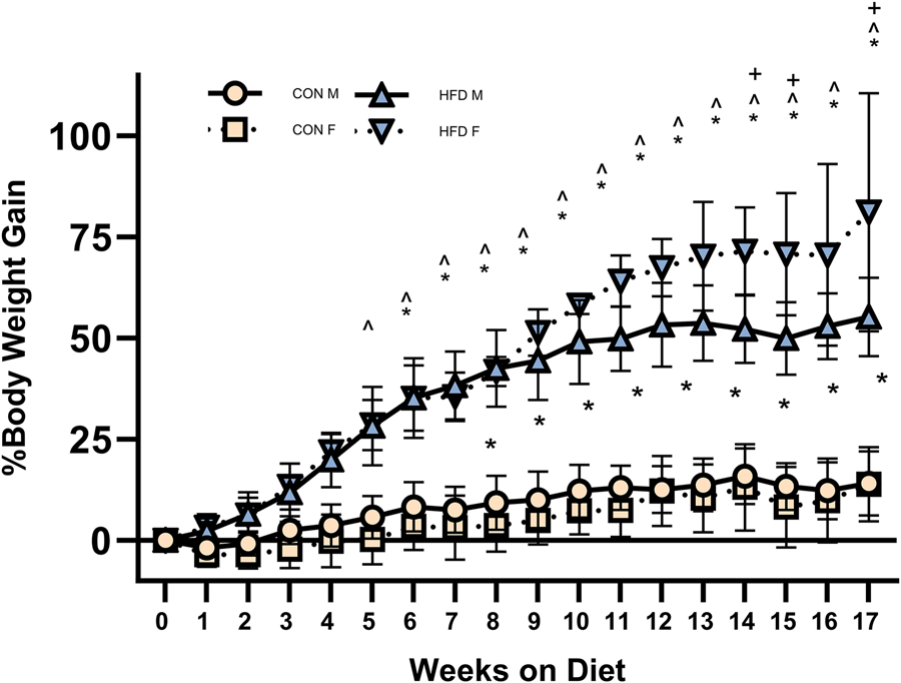
Body weight gain (%) over the duration of the diet. Male and female (M/F) control (CON) and high‐fat diet (HFD) fed groups were weighed weekly, and percentage weight gain was calculated and reported over time. Values reported as means ± SD (*n* = 5–8 per group). Three‐way ANOVA with multiple comparisons (Diet × Sex × Time interaction term, *P *< 0.05): * vs. CON M, ^ vs. CON F, + vs. HFD M, *P* < 0.05).

Due to the brain's dependence on glucose, we tested the circulating metabolism of glucose with a GTT. After fasting, males fed a HFD had a significantly (*P *< 0.05) higher level of baseline glucose and an impaired glucose handling compared to CON males (Figure 2b, c). The females fed a HFD had no differences in fasting glucose levels compared to female controls, yet when challenged during the GTT, an impaired glucose metabolism was identified (Figure 2b, c). Further, the males after a HFD showed more severe impairment of glucose metabolism than the females after a HFD, but both groups after a HFD showed a state of hyperglycaemia.

**FIGURE 2:**
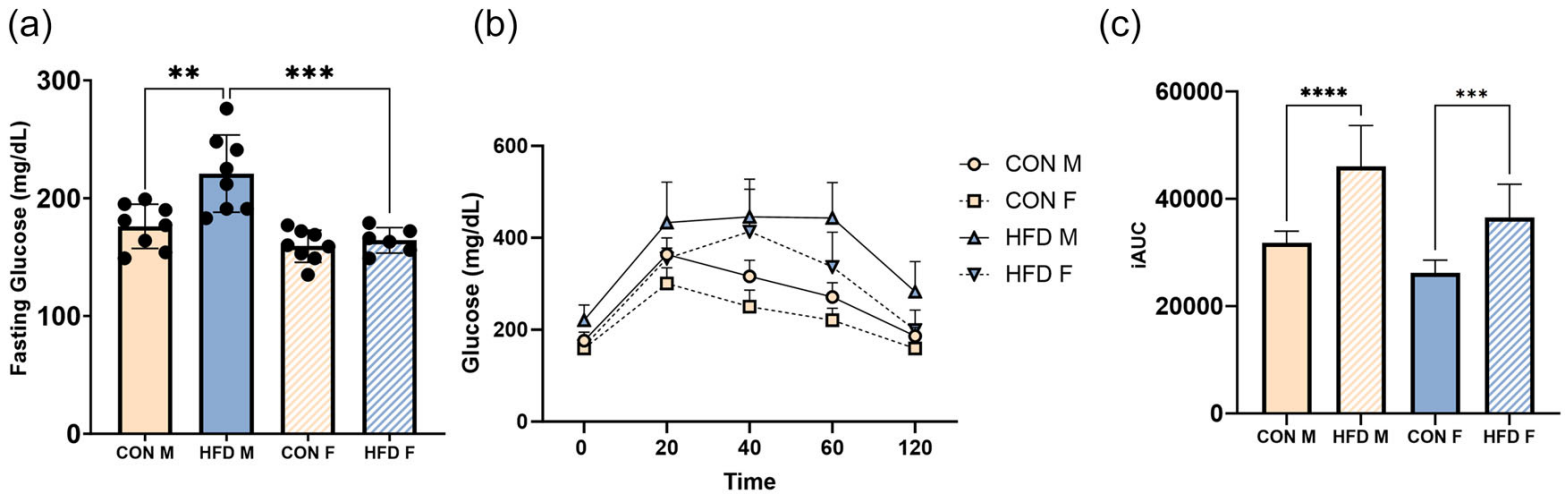
Glucose tolerance test between control (CON) and high‐fat diet (HFD) mice, both male and female (M/F). (a) Fasting glucose levels. (b, c) Glucose levels measured over 120 min and the corresponding incremental area under the curve (iAUC). All values reported as means ± SD (*n* = 6–8 per group). Two‐way ANOVA with multiple comparisons: *P < 0.05.**P<0.01,***P<0.001,****P<0.0001

To further explore the circulating metabolism, we measured insulin and leptin in our animals. Insulin was significantly increased in the HFD male group compared to CON males (*P* = 0.007) (Figure 3), whereas females after a HFD showed no difference in insulin compared to female controls. Leptin was increased in male and female HFD groups compared to their respective controls (*P* < 0.01) (Figure 3).

**FIGURE 3:**
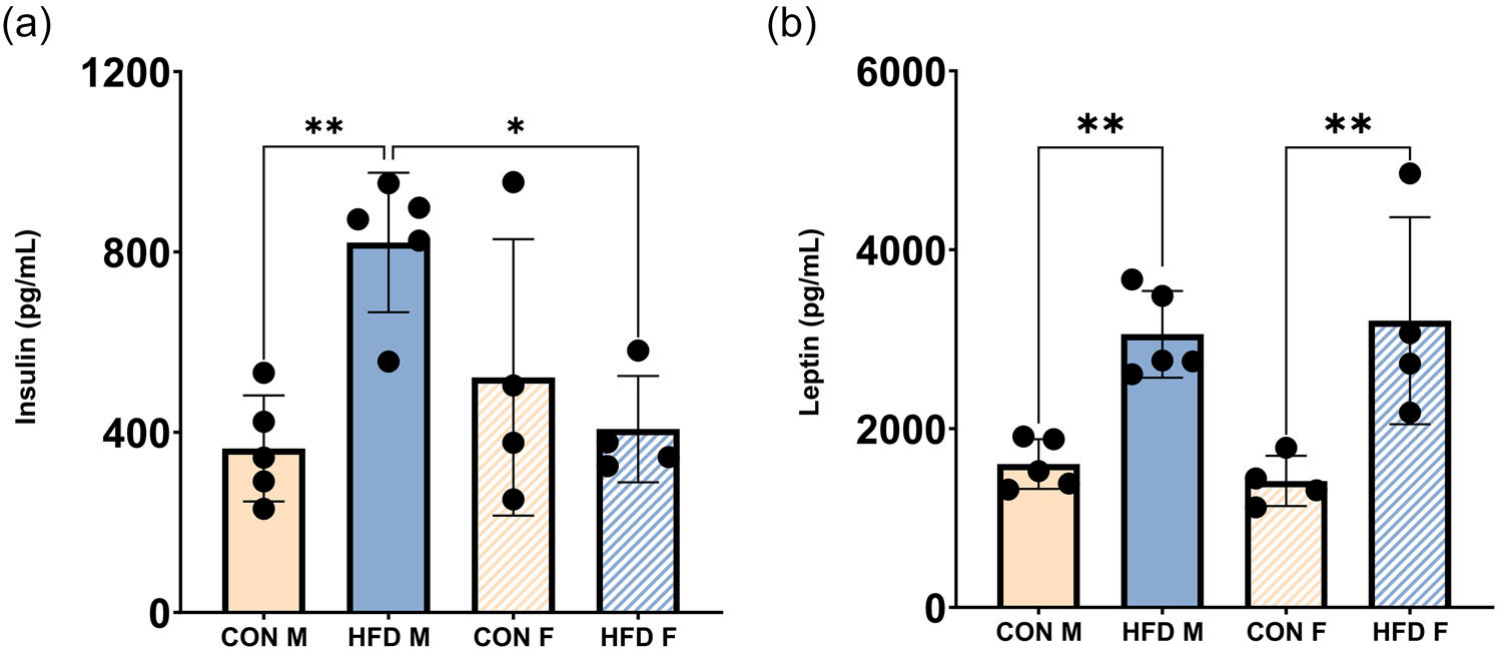
Circulating levels of insulin and leptin. Insulin (a) and leptin (b) in plasma samples between control (CON) and high‐fat diet (HFD) groups. Values reported as means ± SD (*n* = 4–5 per group). One outlier was removed within the HFD F group, and one CON F had undetectable values and was excluded from the analysis. Two‐way ANOVA with multiple comparisons: *P < 0.05, **P < 0.01.

### 3.2 Behavioural outcomes

We performed the open field test to explore anxiety‐like behaviour and locomotion and noted no differences between groups (Figure 4), reflected by similar values for central tendency. However, we noted that the males after a HFD had increased total activity (Figure 4). Next, we performed the Y‐maze test for working memory and tracked spontaneous alternations. No differences between groups were noted, indicating that working memory was not impaired (Figure 4). Total entry into arms was also analysed to ensure no differences between the activity of the mice within the test, and no differences were noted (Figure 4).

**FIGURE 4:**
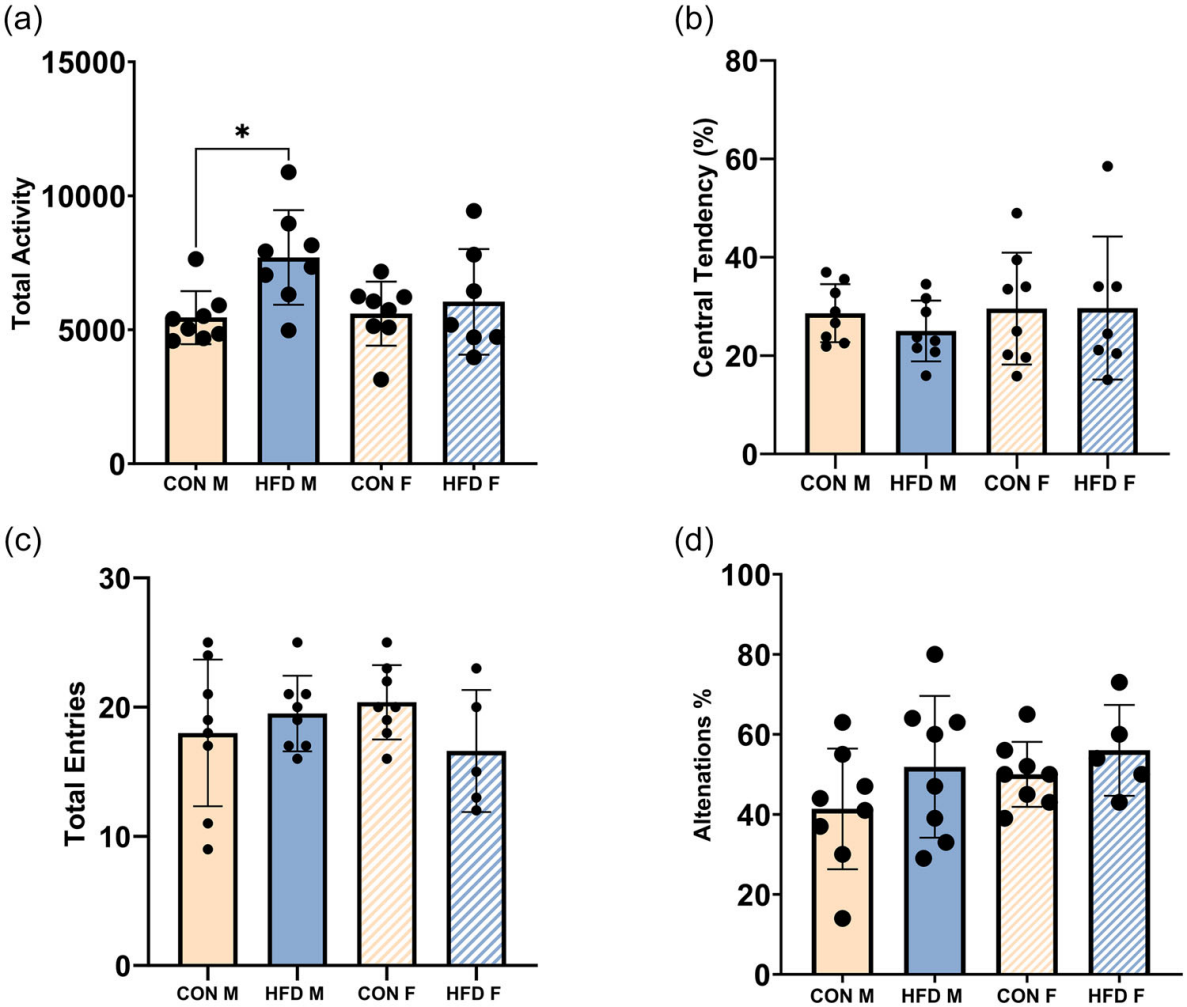
Open field and Y‐maze behavioural tests. (a) Total activity between control (CON) and high‐fat diet (HFD) groups. (b) Central tendency, or anxiety‐like behaviour, between groups. (c) Total entries into the arms of the Y between groups. (d) Percentage of spontaneous alterations between groups. Values reported as means ± SD (*n* = 5–8 per group). Two‐way ANOVA with multiple comparisons: *P < 0.05.

### 3.3 CBF outcomes

Since the brain's metabolism is dependent on the vasculature supplying the necessary nutrients, we explored whether the HFD affected CBF as a measure of relative perfusion. Global CBF was decreased in males after a HFD compared to CON males (*P* = 0.02) (Figure 5). In contrast, females after a HFD showed no differences in global CBF compared to CON females, and no sex differences were noted between the control groups.

**FIGURE 5:**
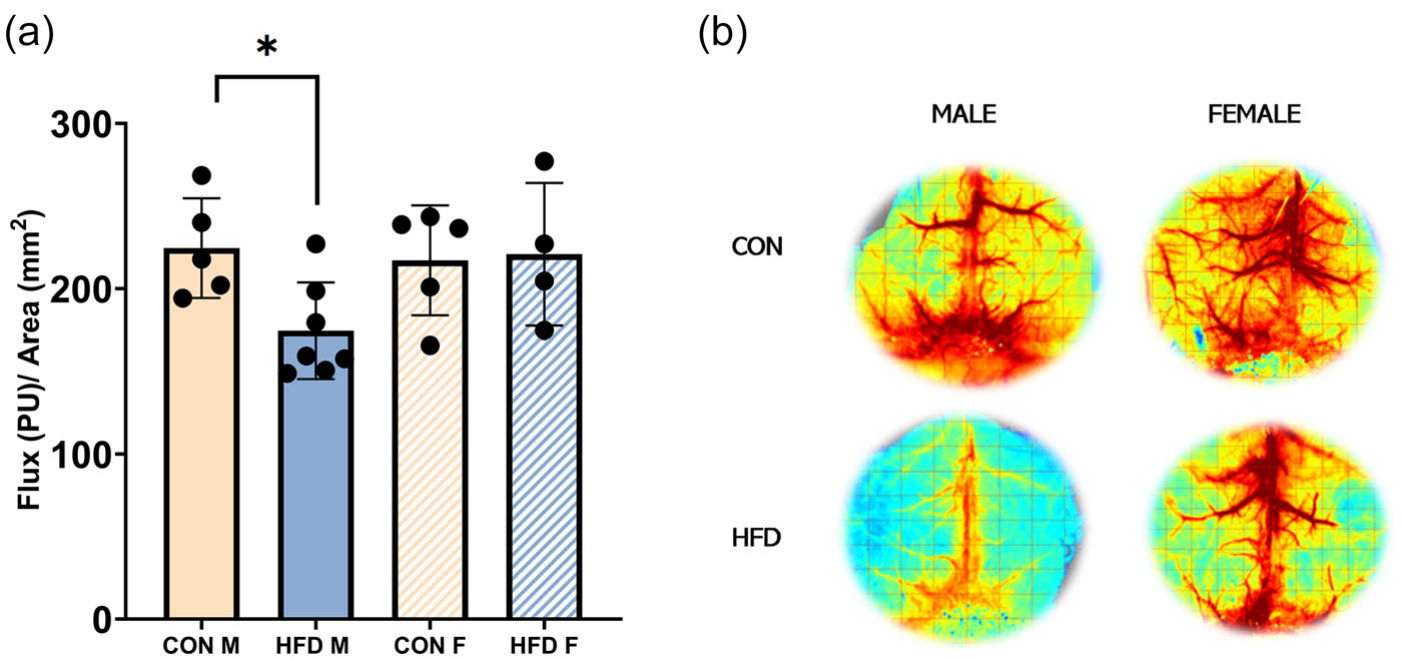
Cerebral blood flux (CBF) measurements. (a) Relative perfusion rates (Flux, PU) were normalized to the area imaged between control (CON) and high‐fat diet (HFD) groups. (b) Representative images of relative perfusion. Values reported as means ± SD (*n* = 4–7 per group). Two‐way ANOVA with multiple comparisons: *P < 0.05.

### 3.4 Mitochondrial substrate utilization and lysine acetylation

Because of the observed impairment of glucose metabolism in the HFD‐fed mice and increased insulin and decreased CBF in males after a HFD, we aimed to understand whether brain mitochondrial substrate utilization was impaired in our HFD‐fed mice. We first assessed the levels of glucose and FAO‐related proteins. Western blot analyses showed a significant decrease in PDH content in CON female brain lysates compared to CON males (*P* = 0.0015) (Figure 6). In contrast, no significant changes were observed in pyruvate carboxylase, suggesting no alterations in glucose oxidation proteins in the brain after a HFD lysate. Examination of FAO proteins LCAD and HADHA (Figure 7) showed no significant changes to HADHA protein content or in LCAD levels within the brain. These findings suggest that overall protein content and levels do not significantly affect fatty acid and glucose utilization. As such, we next sought to address whether mitochondrial electron transport chain (ETC) complexes were impacted in the brain after a HFD.

**FIGURE 6:**
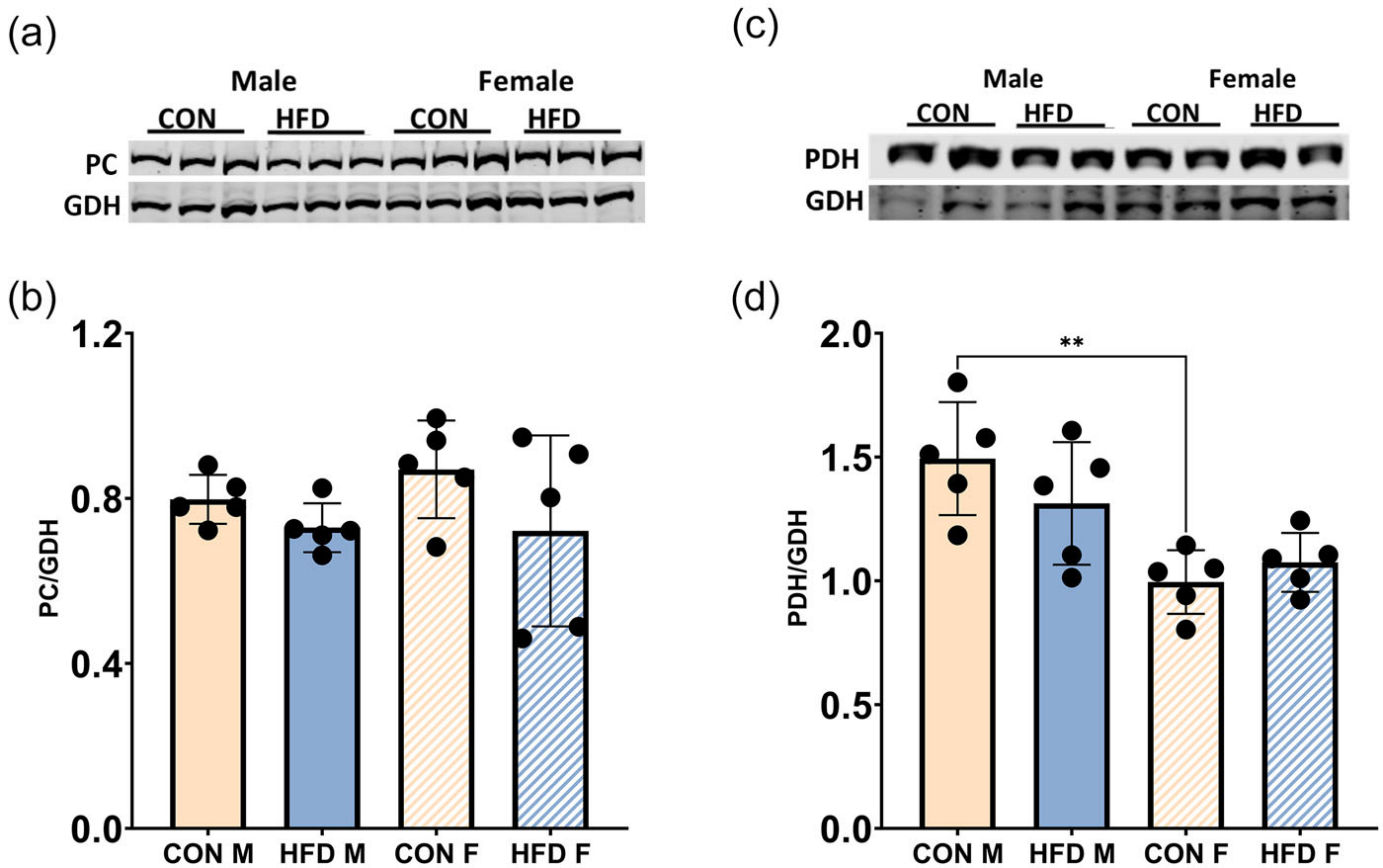
Glucose oxidation markers within the brain. (a) Western blot of brain lysate probed for pyruvate carboxylase (PC) and glutamate dehydrogenase (GDH). (b) Quantification of PC/GDH. (c) Western blot of brain lysate probed for pyruvate dehydrogenase (PDH) and GDH. (d) Quantification of PDH/GDH. Values reported as means ± SD (*n* = 5 per group). Two‐way ANOVA with multiple comparisons: **P < 0.01.

**FIGURE 7:**
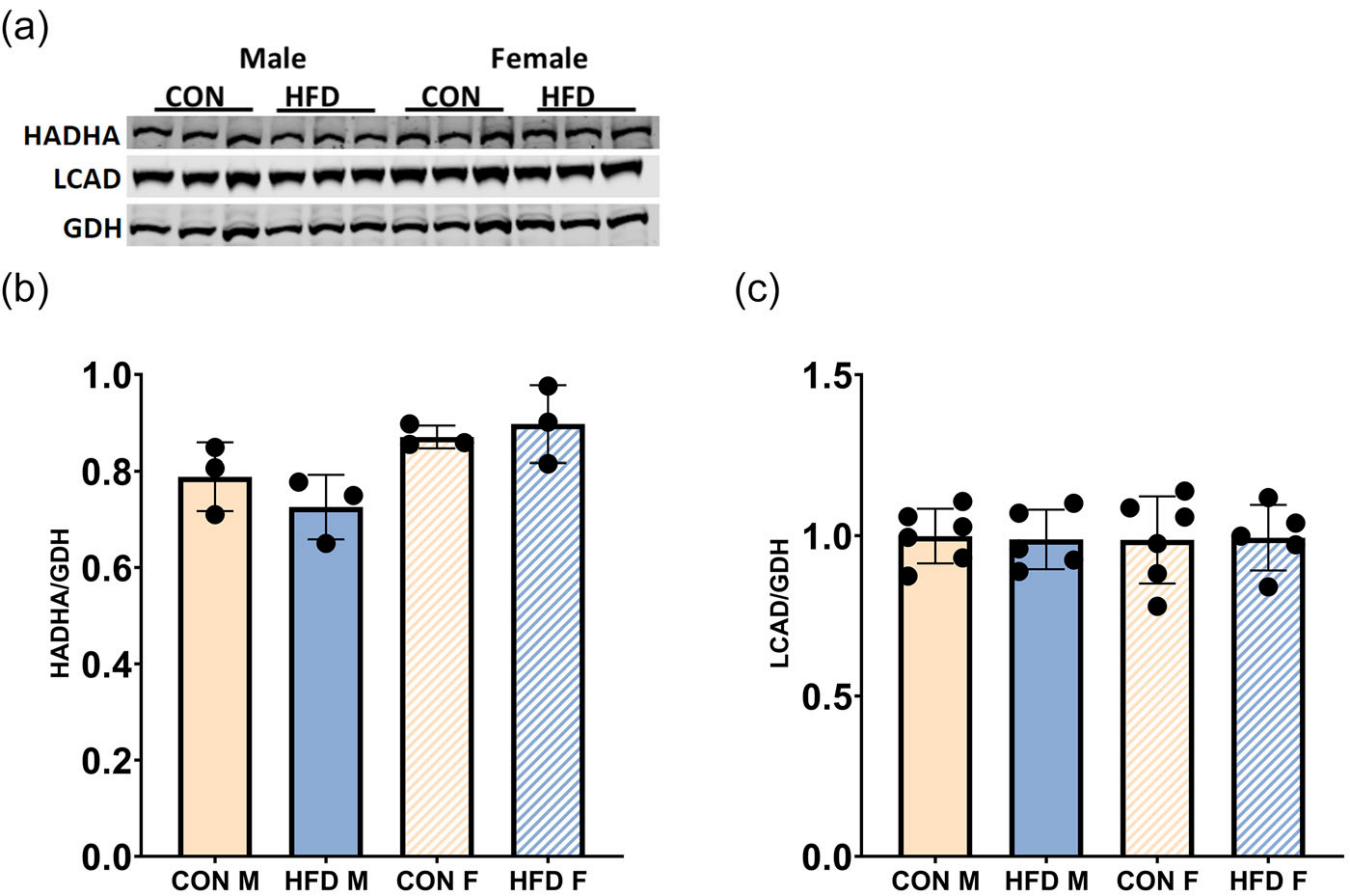
Fatty acid oxidation markers within the brain. (a) Western blot of brain lysate probed for HADHA, LCAD and GDH. (b,c) Quantification of HADHA/GDH (b) and LCAD/GDH (c). Values reported as means ± SD (*n* = 3–5 per group). Two‐way ANOVA with multiple comparisons.

ETC complexes are the primary source of ATP production in mitochondria and are crucial for mitochondrial function. Further, fatty acid and glucose oxidation feed the ETC complex processes. As such, we aimed to investigate whether ETC complex protein content was altered in the brain after a HFD. Utilizing western blot, we assessed one representative protein from each complex, including complex I protein NDUFB8, complex III protein UQCRC2, complex IV protein MTCO1 and complex V protein ATP5A (Figure 8). Our findings showed a significant decrease in NDUFB8 in HFD male samples (*P* = 0.0377) (Figure 8). Further, a significant decrease in UQCRC2 (*P* = 0.047), MTCO1 (*P* = 0.015) and ATP5A (*P* = 0.043) was present in HFD female samples compared to their respective controls (Figure 8). These findings suggest dysfunction within ETC complexes within the brain after a HFD samples independent of sex.

**FIGURE 8:**
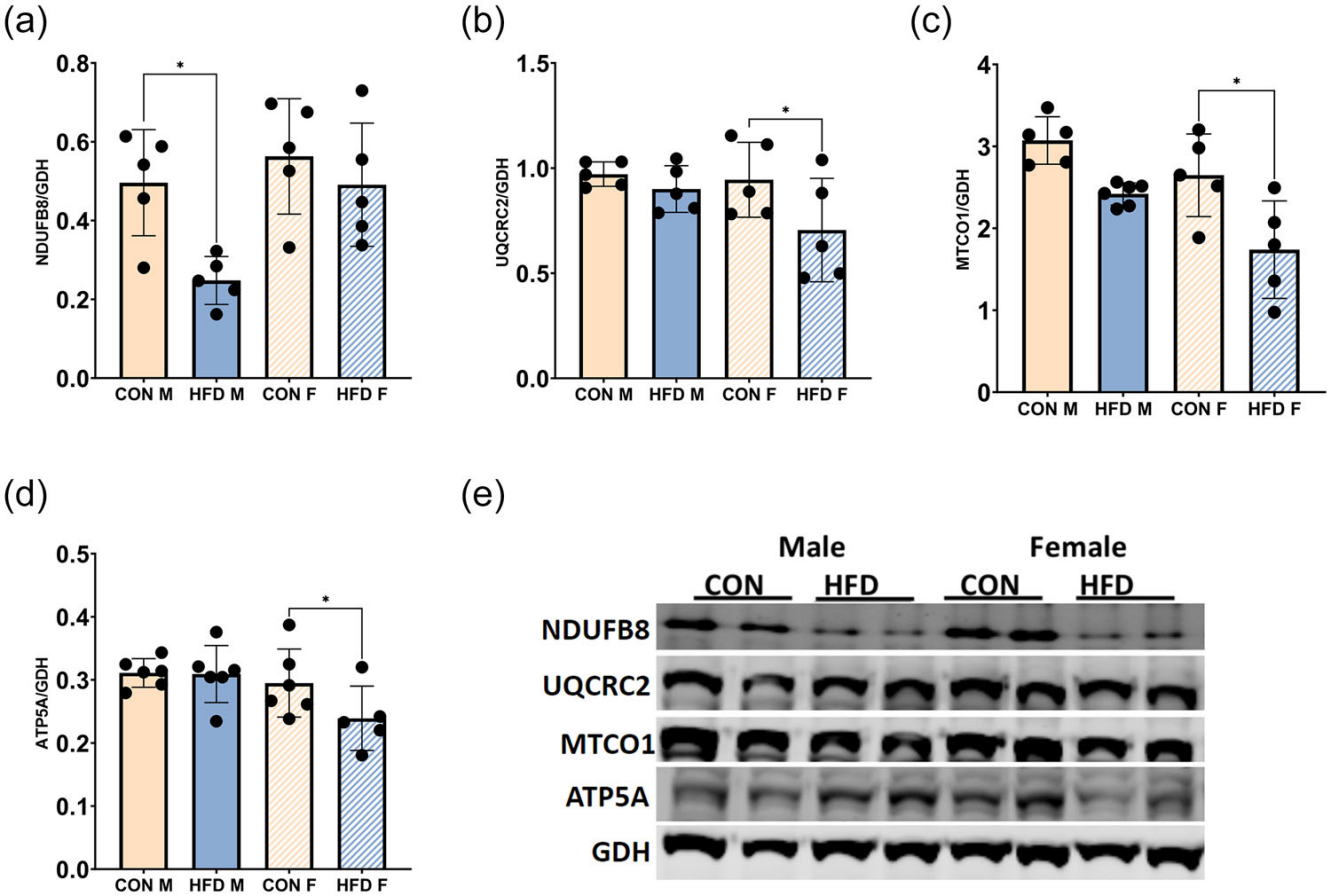
Electron transport chain (ETC) subunits within the brain. Western blot of brain lysates probed for mitochondrial ETC complex subunits. (a–d) Control (CON) and high‐fat diet (HFD) brain lysate quantification of NDUFB8/GDH (a), UQCRC2/GDH (b), MTCO1/GDH (c) and ATP5A/GDH (d) between groups. Values reported as means ± SD (*n* = 5 per group). Two‐way ANOVA with multiple comparisons: *P < 0.05. (e) Western blot of brain lysates probed for NDUFB8, UQCRC2, MTCO1, ATP5A and GDH.

The role of lysine acetylation in regulating mitochondrial fatty acid, glucose oxidation and ETC complex protein function has been previously reported in HFD hearts. As such, we investigated whether lysine acetylation also plays a regulatory role in the brain. We assessed total brain protein acetylation levels using a pan acetyl‐lysine antibody and observed a significant decrease in acetylation of total brain proteins in males after a HFD compared to CON males (*P* = 0.029) (Figure 9). However, we observed a significant increase in brain protein acetylation in HFD female samples compared to their controls (*P* = 0.012). These findings suggest that sex‐ and diet‐dependent changes in overall brain protein acetylation may impact its function. We further explored whether the acetylation status of previously reported fatty acid and glucose oxidation proteins, as well as ETC complex proteins, was affected in our study. Immunoprecipitation pulldown with a pan acetyl‐lysine antibody presented similar changes in the acetylation of these proteins as observed in total protein content. LCAD, PDH and PC were all hyperacetylated in the HFD female brain, whereas a decrease in acetylation level was observed in the HFD male group. Similarly, acetylation levels of NDUFB8 and MTCO1 were decreased in HFD male brains and increased in HFD female samples. These findings suggest that mitochondrial lysine acetylation potentially plays a crucial role in regulating substrate utilization and ETC complex function in a HFD brain.

**FIGURE 9:**
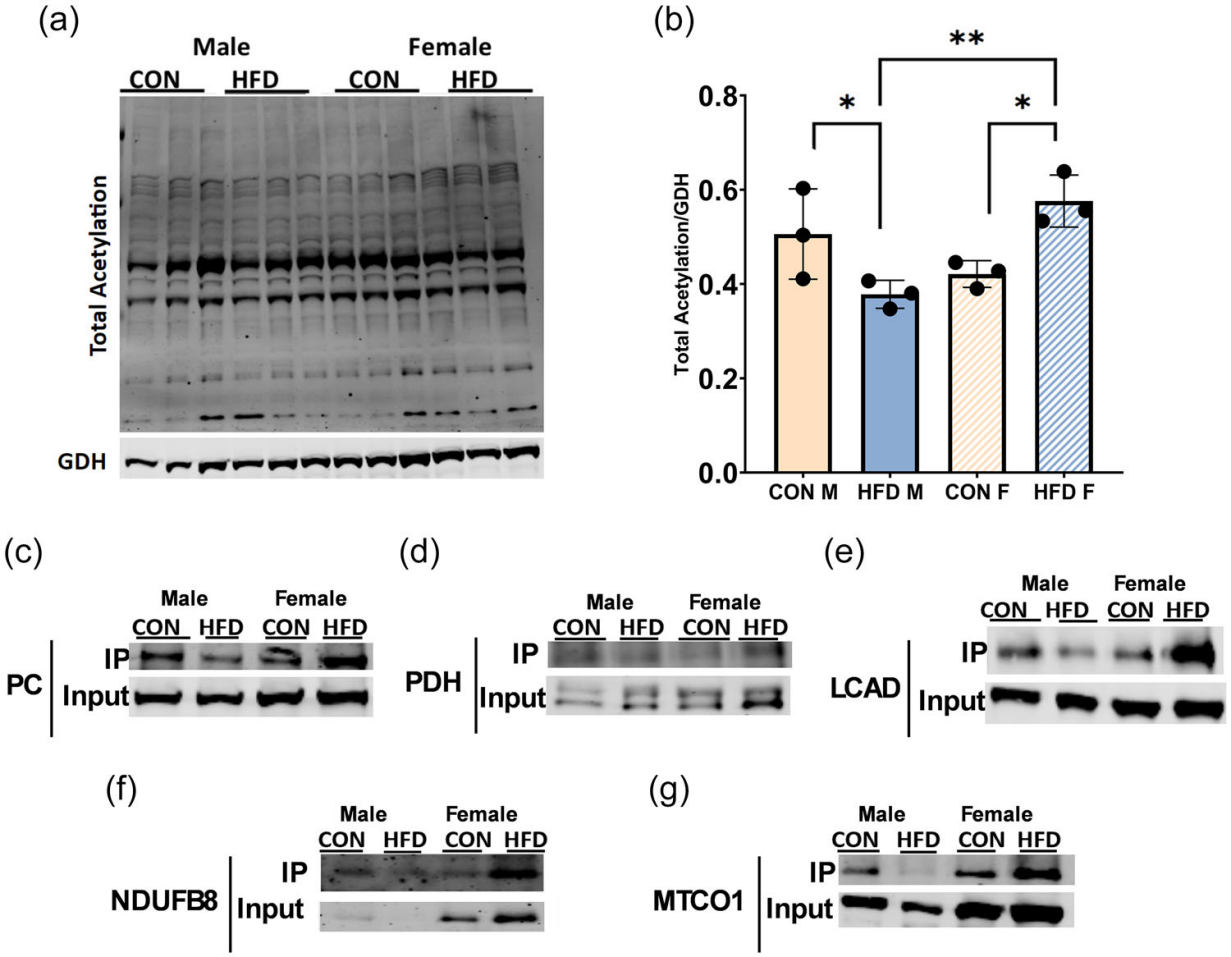
Lysine acetylation within the brain. Western blot of brain lysate probed for pan acetyl‐lysine between groups. (b) Quantification of Total acetylation/GDH between groups. Values reported as means ± SD (*n* = 3 per group). Two‐way ANOVA with multiple comparisons: *P < 0.05, **P < 0.01. (c–g) Immunoprecipitation pulldown and input of brain lysate probed for PC, PDH, LCAD, NDUFB8 and MTCO1.

## 4 DISCUSSION

The goal of this study was to assess how cerebrovascular health, cognitive effects and mitochondrial metabolism were impacted by a HFD and whether lysine acetylation, a post‐translational modification shown to regulate several mitochondrial processes, played a role in regulating brain metabolism. Whilst no differences were observed in behavioural and cognitive function, our novel findings suggest a sex‐dependent role of lysine acetylation in regulating glucose oxidation and FAO, as well as mitochondrial ETC complexes. We show that HFD‐induced obesity resulted in increased lysine acetylation in the female brains after a HFD compared to CON females. In contrast, a significant decrease in lysine acetylation was observed in the male brains after a HFD compared to CON males. This striking difference in acetylation was also evident in acetylation levels of fatty acid oxidation, glucose oxidation and ETC complex I and IV proteins. Finally, we also show that the cerebral perfusion rate was decreased in males after a HFD, but not in females, suggesting reduced substrate availability, potentially leading to observed sex differences in protein acetylation in the brain.

As expected, we noted increased adiposity and glucose metabolism between the HFD and CON groups, independent of sex. Increases in resting blood glucose showed a state of hyperglycaemia in males after a HFD. However, both males and females, after a HFD, showed impaired glucose metabolism when challenged. This agrees with previous literature that reported a state of hyperglycaemia in both sexes (Gallou‐Kabani et al., 2007; Salinero et al., 2018). Males after a HFD also had higher insulin levels, further indicating an impaired state of glucose metabolic function. Additionally, we found that females after a HFD proportionally gained more weight than males after a HFD, commonly not noted in younger mice, as oestrogen is suggested to have a protective effect on obesity and glucose sensitivity in young female mice (Dakin et al., 2015; Stubbins et al., 2012). However, in more middle‐aged phenotypes, female mice have been noted to gain more weight, which we were able to replicate within our study (Salinero et al., 2018). These weight changes were further characterized by decreased lean mass whilst fat mass increased in HFD‐fed groups. Additionally, mice after a HFD had increased leptin levels, leading to increased caloric consumption, commonly reported in a HFD model (R. Zhang et al., 2016).

This state of hyperglycaemia and weight gain can lead to several metabolic syndromes. Within human patients, CBF is significantly impaired in obese populations (Dorrance et al., 2014), and in a rodent model of metabolic syndrome, we have shown that cerebrovascular endothelial function and wall mechanics are impaired in obese male Zucker rats (Brooks et al., 2015). Moderate deficits in CBF can lead to reductions in ATP synthesis and have been commonly linked to Alzheimer's and vascular dementia (Kivipelto et al., 2005; Kalaria, 2010). Within our current study, we noted that males after a HFD had a reduced relative perfusion rate. This agrees with our previously published functional data showing impaired cerebrovascular function and structural changes in the isolated middle cerebral artery (MCA) (Brooks et al., 2015). However, in this study, HFD‐fed female mice had no change in relative cerebral perfusion; however, on average, females had increased CBF compared to males, which has been reported in mice and humans (Easson et al., 2023; Raman‐Nair et al., 2024). We have also previously shown that obese female Zucker rats had significant endothelial dysfunction of the MCA compared to lean females and that the magnitude of this endothelial dysfunction was substantially smaller compared to what was observed between lean and obese male Zucker rats (Brooks et al., 2018). Furthermore, when female obese rats underwent an ovariectomy to study the effects of oestrogen deficiency, they had a substantial reduction in MCA endothelial function similar to what was noted between male obese and lean rats. These data may suggest that female mice in our study may still have some protective effects of oestrogen on cerebrovascular function and relative perfusion.

Whilst systemic insulin increases peripheral blood flow, its effect on the brain is more complex because insulin does not stimulate brain glucose metabolism as it does in muscle, liver and fat (Agrawal et al., 2021). The cerebral endothelial cells do have insulin receptors (Dorrance et al., 2014) and promote vasodilatation in the isolated MCA (Katakam et al., 2009; Olver et al., 2018). However, no such response is noted when insulin is systemically infused in rats (Olver et al., 2017), and human studies have reported variable results (Chaudhuri et al., 1999; Grichisch et al., 2012; Kim et al., 2007; Schilling et al., 2014). Two recent studies have reported that peripheral insulin resistance was associated with reduced cerebral blood flow (Deery et al., 2024; Han et al., 2023). Further, Shariffi et al. (2025) showed that insulin administered systemically (intravenous) or locally (intranasal) did not affect the blood velocity in the MCA of young healthy adults. In contrast, insulin reduced cerebrovascular compliance (a measure of changes in vascular tone) regardless of the administration method, suggesting insulin‐mediated vasorelaxation. Women have also been shown to be less prone to insulin resistance in muscle tissue when compared to men (Høeg et al., 2011). Given that cerebral hypoperfusion is prevalent with obesity (Toda et al., 2014) and possibly connected to low brain insulin concentrations (Kaiyala et al., 2000), it is interesting to note that in our study, the female mice after a HFD are not impaired in CBF and do not have increased insulin in comparison to their CON counterparts. This could also imply a sex‐specific role within our male mice on a HFD, having decreased CBF values, which could potentially be due to insulin resistance. Future work needs to tease out this relationship between brain insulin resistance and CBF in the context of HFD.

Within our study, we noted differences in circulating glucose levels, indicating metabolic disruption. As such, we explored how proteins related to glucose oxidation were impacted in our model. We observed no changes in total PC or PDH within the brain between diets. However, we noted an increase in PDH levels in female mice. Aged female mice, ∼20 months old, have an increase in PDH compared to their male counterparts, which diminishes within an ovariectomy model, suggesting a potential role of oestrogen in glucose metabolism (Gaignard et al., 2015). With no changes in protein content of the glucose oxidation process, we assessed the potential role of acetylation in regulating the activities of these proteins. Lysine acetylation has been shown to regulate the activities of proteins related to mitochondrial metabolism (De Marchi et al., 2019; Thapa et al., 2017). The overall acetylation profile in the brain after an HFD showed a sex‐specific effect, as observed by decreased acetylation in males after a HFD and increased acetylation status in HFD female brains. Acetylation has been shown to regulate the activity of PDH in the HFD heart (Thapa et al., 2022). Indeed, our acetyl‐lysine immunoprecipitation data also show sex‐specific changes in acetylated PDH levels, suggesting a potential role of acetylation in overall glucose activity.

Whilst glucose is the brain's primary fuel, FAO can contribute significantly to brain function. FAO has been reported to have a preservative effect on the mitochondrial respiratory chain within the brain (Morant‐Ferrando et al., 2023). Additionally, others have reported that the brain can use other energy sources like fatty acids or ketones (Bélanger et al., 2011). However, due to the glycolytic reliance of the brain, the role of FAO in the brain's energy supply is not as well known. Based on the observed glucose impairment, we assessed whether FAO was impacted in our model. No differences in LCAD and HADHA protein expression were observed. However, acetylation levels of LCAD, which have been previously reported to regulate its activity in the HFD heart, showed sex‐specific alterations in our model.

Lactate is another competing fuel source for the brain, and obesity has been shown to increase fasting blood lactate levels within humans (Crawford et al., 2008; Reaven et al., 1988), which may reflect an imbalance between lactate production via glycolysis and its clearance via mitochondrial oxidation. Indeed, high blood lactate has been suggested as a biomarker for mitochondrial dysfunction and metabolic imbalance (Shayota, 2024). However, other studies (Tsai, Chen et al., 2018, 2018) have reported reduced brain (hippocampus) lactate levels in mice exposed to a HFD. Lactate is primarily produced by astrocytes, whereby astrocytes take up glucose and convert it into lactate to shuttle to neurons. The importance of lactate and its relationship with mitochondrial disturbances is reflected by some evidence suggesting that lactate could be converted to lactyl‐CoA, which acts as a precursor for the post‐translational modification of lysine residues on proteins, including histones (Lu et al., 2024; Niu et al., 2024; D. Zhang et al., 2019). As such, future research should explore this relationship as it relates to obesity and the development of dementia.

Cerebral vascular dysfunction, observed in our males after a HFD, can lead to varying degrees of cognitive decline and metabolic dysregulation, which have been postulated to result from mitochondrial dysfunction (Huang et al., 2023; Verdelho et al., 2021). To further explore the role mitochondrial dysfunction may have on cerebral vascular dysfunction, we measured protein expressions of components within ETC complexes. Examination of the ETC complex protein content showed a significant decrease in complex I protein NDUFB8 in males after a HFD. A decrease in complex III protein UQCRC2, complex IV MTCO1 and complex V ATP5A was observed in the female HFD‐fed group compared to controls. These observations showed metabolic disruption in oxidative respiration within both sexes exposed to a HFD. To further explore these changes, we observed acetylation levels of complex I and IV proteins, resulting in decreased acetylation levels in males after a HFD and increased levels in females after a HFD. Coupled with decreased expression of these proteins, our findings suggest a functional and structural disruption in the ETC process, a critical contributor to mitochondrial dysfunction. Previous work has also highlighted mitochondrial dysfunction within the HFD‐fed brain, noting a cycle between mitochondrial dysfunction and an oxidative environment due to an obese state (Cavaliere et al., 2019; Langley et al., 2020). This pro‐oxidative environment, which damages mitochondria, also creates a pro‐inflammatory state. Ultimately, the combination of these factors is what commonly links obesity to cognitive impairments. As such, further investigation of how acetylation regulates the oxidative environment in the brain after a HFD is warranted.

Additionally, this study at present did not investigate the changes within specific brain regions (i.e., cortex, hypothalamus, hippocampus). All of which have been shown to play key roles in learning and memory within dementia progression (Haroutunian et al., 2009; Samuel et al., 1991; Suzuki et al., 2019). Future studies should investigate the role of substrate utilization within specific brain regions to better understand the underlying mechanisms of neurological diseases.

The diet‐induced obesity alters cerebrovascular health in male mice, indicated by hypoperfusion, coupled with decreases in acetylation of proteins involved in mitochondrial and bioenergetic pathways. However, in female mice, no differences were noted in relative cerebrovascular perfusion rates, and the opposite resulted in increased acetylation in bioenergetic proteins. One possible explanation for why we saw such striking sex differences could potentially be explained by perfusion rates. An impairment in either energy metabolism or cerebral hypoperfusion will result in a deficit of the other (Schulz et al., 2009; Venkat et al., 2016). Keeping this in mind, the increases in circulating FFA known to be caused by a HFD, coupled with decreases in perfusion within the brain, stunting the ability to deliver substrate for that process, could cause increases in acetylation in females after a HFD. However, substrate availability could be lowered within males due to reduced perfusion, decreasing acetylation. Our data complement the growing body of literature coupling the effect CBF and metabolic demand have on each other within the brain, emphasizing the importance of investigating these findings further (Paulson et al., 2010).

Even though we reported significant changes in cerebral and metabolic health within our study, these effects did not affect working memory or anxiety‐like behaviour within our groups. Previous studies have established cognitive decline in learning and memory with aged mice on a HFD compared to younger mice fed a HFD (Henn et al., 2022; Tucsek et al., 2014). Our results with mice at 10 months of age support the current literature that HFD feeding only shows cognitive impairment in more advanced ages, typically in mice aged 12–24 months. In addition, we saw no decreases in total activity within our mice on a HFD over the 30‐min open field testing, indicating our groups on a HFD were not physically impaired. As overall activity levels could confound the data within the groups on a HFD, future studies should also consider more thorough measures, such as wheel‐running or CLAMS, to compare the energy expenditure of groups.

In summary, our findings propose lysine acetylation as a novel and potential regulatory mechanism that impacts vascular and metabolic function in the brain mitochondria. Specifically, the impact of acetylation observed in mitochondrial glucose oxidation, FAO and ETC complex proteins provides a new target to attenuate mitochondrial dysfunction in the HFD‐induced obesity model. The sex‐specific differential changes observed in total acetylation levels of male and female brains after a HFD present a unique opportunity to further investigate these processes by comparing a younger and older mouse model. Our future studies will also focus on utilizing acetylation and deacetylation animal models further to delineate the impact of acetylation on diet‐induced obesity and brain function.

## AUTHOR CONTRIBUTIONS

The study was performed in Paul D. Chantler's and Dharendra Thapa's laboratories. *Conceived and designed research*: Paul D. Chantler, Dharendra Thapa, Eric E. Kelley, Randy W. Bryner, Kate Karelina, Nicole N. Eminhizer and Alec Hanshew. *Performed experiments*: Nicole N. Eminhizer, Dharendra Thapa, Jackson Stewart, Alec Hanshew, Steven Ball and Saina S. Prabhu. *Analysed data*: Paul D. Chantler, Nicole N. Eminhizer and Christa Lilly. *Interpreted results of experiments*: Paul D. Chantler, Dharendra Thapa and Nicole N. Eminhizer. *Prepared figures*: Paul D. Chantler, Dharendra Thapa and Nicole N. Eminhizer. *Drafted manuscript*: Nicole N. Eminhizer. *Edited and revised manuscript*: Paul D. Chantler, Dharendra Thapa and Nicole N. Eminhizer. All the authors have reviewed the final version. All authors have read and approved the final version of this manuscript and agree to be accountable for all aspects of the work in ensuring that questions related to the accuracy or integrity of any part of the work are appropriately investigated and resolved. All persons designated as authors qualify for authorship, and all those who qualify for authorship are listed. During the preparation of this work the author(s) did not use any AI technologies in the writing process.

## CONFLICT OF INTEREST

The authors declared no potential conflicts of interest with respect to the research, authorship, and/or publication of this article.

## Data Availability

The data supporting the findings of this study are available on request from the corresponding author.
